# Emerging connections between GPI-anchored proteins and their extracellular carriers in colorectal cancer

**DOI:** 10.20517/evcna.2023.17

**Published:** 2023-05-18

**Authors:** Oleg S. Tutanov, Sarah E. Glass, Robert J. Coffey

**Affiliations:** ^1^Department of Medicine, Vanderbilt University Medical Center, Nashville, Tennessee 37232, USA.; ^2^Department of Cell and Developmental Biology, Vanderbilt University, Nashville, Tennessee 37232, USA.; ^#^Authors contributed equally.

**Keywords:** Extracellular vesicles, exosomes, exomeres, supermeres, GPI-anchored proteins, biomarkers, colorectal cancer

## Abstract

Although extracellular vesicles (EVs) were discovered over 40 years ago, there has been a resurgence of interest in secreted vesicles and their attendant cargo as novel modes of intracellular communication. In addition to vesicles, two amembranous nanoparticles, exomeres and supermeres, have been isolated and characterized recently. In this rapidly expanding field, it has been challenging to assign cargo and specific functions to a particular carrier. Refinement of isolation methods, well-controlled studies, and guidelines detailed by Minimal Information for Studies of Extracellular Vesicles (MISEV) are being employed to “bring order to chaos.” In this review, we will briefly summarize three types of extracellular carriers - small EVs (sEVs), exomeres, and supermeres - in the context of colorectal cancer (CRC). We found that a number of GPI-anchored proteins (GPI-APs) are overexpressed in CRC, are enriched in exosomes (a distinct subset of sEVs), and can be detected in exomeres and supermeres. This affords the opportunity to elaborate on GPI-AP biogenesis, modifications, and trafficking using DPEP1, a GPI-AP upregulated in CRC, as a prime example. We have cataloged the GPI-anchored proteins secreted in CRC and will highlight features of select CRC-associated GPI-anchored proteins we have detected. Finally, we will discuss the remaining challenges and future opportunities in studying these secreted GPI-APs in CRC.

## INTRODUCTION

Cells release a dizzying array of EVs and nanoparticles, which differ in size, biogenesis, function, and cargo, as has been recently reviewed by our group^[1]^. In the present review, we have chosen to focus on three subsets of EVs and nanoparticles: exosomes, exomeres, and supermeres, as they have the most relevance to GPI-AP cargo. Exosomes, ranging in size from 40 - 150 nm, are a subset of lipid bilayer-enclosed small extracellular vesicles (sEVs). They are defined as being of endocytic origin and containing tetraspanins, CD9, CD63, and CD81^[2,3]^. Herein, we will use the more inclusive term, sEVs, unless the classic criteria for exosomes are met. sEVs are increasingly recognized for their role in intercellular communication and their cargo as potential diagnostic biomarkers and therapeutic targets^[4,5]^. Over the years, numerous studies have shown that sEVs play an important role in transferring functional proteins and nucleic acids in both health and disease^[6]^. They have been implicated in various oncogenic processes such as immunosuppression, immune evasion, angiogenesis, epithelial-to-mesenchymal transition, establishment of a pro-tumorigenic microenvironment and metastatic niche, and drug resistance^[5,7,8]^. The composition and content of sEVs can reflect the status of the cell of origin, making the analysis of sEVs an area of interest for the development of non-invasive diagnostic tools^[9]^. In addition to their diagnostic potential, sEVs are also being studied as therapeutic targets, with research exploring the possibility of blocking or modulating sEV release as a strategy for cancer treatment^[10]^. The recent discovery of exomeres and supermeres offers new opportunities for further clinical exploration and translation^[8,11]^.

Exomeres, nanoparticles with a size range of 30 - 60 nm, were first described by Lyden et al. in 2017^[11]^ and are a relatively new addition to the collection of extracellular particles. Unlike EVs, exomeres are thought to lack a double-lipid membrane, but they are associated with a unique set of RNAs and proteins^[11]^. The potential functions of exomeres are still being investigated, but they have been shown to play a role in intercellular communication and regulation of cellular processes^[12,13]^. For example, exomeres have been implicated in modulating signaling pathways, cell adhesion, and immune responses in recipient cells^[13]^.

Supermeres, nanoparticles of an even smaller size than exomeres (25 - 35 nm), were first described in a recent study from our lab and are another addition to the world of extracellular nanoparticles^[13]^. First found in a CRC cell line, supermeres are morphologically and structurally distinct from exomeres and have different cellular uptake kinetics compared to sEVs and exomeres *in vitro* as well as greater uptake *in vivo*^[13]^. Supermeres have been found to contain high levels of clinically relevant proteins such as APP, MET, GPC1, AGO2, and TGFBI, which were previously reported to be present in exosomes^[13]^. Furthermore, most of the extracellular RNA (exRNA) was found to be associated with supermeres rather than sEVs and exomeres^[13]^. There are concerns that similarities between exomeres and supermeres suggest a continuum of nanoparticles only differing in size, but we contend that distinct differences in size, structure, cargo, and biological properties warrant their separate classification at this time^[14]^. Therefore, we propose that exosomes, exomeres, and supermeres are distinct subtypes of circulating EVs and nanoparticles (EVPs). By examining these subtypes as separate entities, we can gain a more complete understanding of EVP biogenesis, their individual biological effects, and the overall interplay, which can be more easily achieved now after our publication of comprehensive isolation method of EVs, exomeres, and supermeres from the same stating material^[15]^. These discoveries can translate to elucidating the roles these secreted particles play in remodeling the tumor microenvironment, invasion, immune suppression, and other processes that are important for colorectal carcinogenesis.

One way to understand the role of various EVP classes is by assessing their cargo. Glycosylphosphatidylinositol-anchored proteins (GPI-APs) are a distinctive subclass of EV-associated proteins due to their unique GPI-anchor and enrichment in EVs in comparison to cells, as is explained by their affinity for membranes and lipid rafts^[16,17]^. GPI-APs are elevated in the blood of patients with CRC in comparison to healthy individuals^[18]^. Some GPI-APs, such as CEACAM5 and CD73, are known to be enriched in EVs, highlighting their use as CRC biomarkers^[19,20]^. Interestingly, some GPI-APs are also enriched in exomere and supermere fractions (e.g., GPC1). GPI-APs are being investigated as immunotherapy targets and the GPI-anchor has been used as a novel biological modification for EV content on the surface when therapeutically targeting tumor cells^[21-23]^. The presence of GPI-APs in various EVP fractions has important implications for the way cells communicate and regulate signaling pathways. It also offers insight into the biogenesis, secretion, and interaction of various EVP subpopulations.

## GPI BIOGENESIS, SORTING, AND RELEASE

Before discussing specific GPI-linked cargo present in CRC EVPs, it is helpful to review key features of GPI biogenesis, sorting, and release. To do this, we will feature dipeptidase-1 (DPEP1), one of the most abundant GPI-APs in CRC exosomes, as an illustrative example^[13,24]^. For those interested in a historical perspective on GPIs, there have been several excellent recent reviews^[25,26]^. GPI-APs are proteins covalently attached post-translationally at the C-terminus to glycosylated phosphatidylinositol that allows for membrane anchorage^[26]^. GPIs in various organisms have a common backbone consisting of ethanolamine phosphate (EtNP), three mannoses (Mans), one non-N-acetylated glucosamine (GlcN), and inositol phospholipid, whose structure is EtNP-6Manα-2Manα-6Manα-4GlNα-6myo inositol-P-lipid with the lipid moiety being either phosphatidylinositol of diacyl or 1-alkyl-2-acyl form, or inositol phosphoceramide^[27-29]^. Currently, there are 140 reviewed human GPI-APs listed in the Uniprot database^[30]^, of which 108 have been reported as cargo in EVs in Vesiclepedia^[31]^ and 66 have been identified as cargo in EVs specifically derived from CRC cells [Table 1]. These 140 annotated GPI-APs are enriched for proteins facilitating immune response and cell-cell communication, with > 40 of the GPI-APs displaying different enzymatic activities^[26]^.

**Table 1:** Complete list of currently reviewed human GPI-APs, their presence in EVs in general and in CRC EVs in particular (in bold)

**Gene Name**	**Protein Name**	**Vesiclepedia**
ACHE	Acetylcholinesterase	+
ALPG	**Alkaline phosphatase, germ cell type**	**+**
ALPI	**Intestinal-type alkaline phosphatase**	**+**
ALPL	**Alkaline phosphatase, tissue-nonspecific isozyme**	**+**
ALPP	**Alkaline phosphatase, placental type**	**+**
ART1	GPI-linked NAD(P)(+)--arginine ADP-ribosyltransferase 1	
*ART3^EV^^,^^ EX^^,^^ S^*	*Ecto-ADP-ribosyltransferase 3*	*+*
ART4	Ecto-ADP-ribosyltransferase 4	+
BCAN	Brevican core protein	+
BST1	**ADP-ribosyl cyclase/cyclic ADP-ribose hydrolase 2**	**+**
BST2	**Bone marrow stromal antigen 2**	**+**
CA4	Carbonic anhydrase 4	+
*CD109 ^EV^^,^^ EX^^,^^ S^*	***CD109 antigen***	***+***
CD14	**Monocyte differentiation CD14 antigen**	**+**
CD160	CD160 antigen	
CD177	CD177 antigen	+
CD24	Signal transducer CD24	+
CD48	CD48 antigen	+
CD52	CAMPATH-1 antigen	+
*CD55 ^EV^^,^^ S^*	***Complement decay-accelerating factor***	***+***
*CD59 ^EV^^,^^ EX^*	***CD59 glycoprotein***	***+***
CDH13	**Cadherin-13**	**+**
*CEACAM5 ^EV^^,^^ EX^^,^^ S^*	***Carcinoembryonic antigen-related cell adhesion molecule 5***	***+***
*CEACAM6 ^EV^^,^^ EX^^,^^ S^*	***Carcinoembryonic antigen-related cell adhesion molecule 6***	***+***
CEACAM7	**Carcinoembryonic antigen-related cell adhesion molecule 7**	**+**
CEACAM8	**Carcinoembryonic antigen-related cell adhesion molecule 8**	**+**
CFC1	Cryptic protein	
CNTFR	Ciliary neurotrophic factor receptor subunit alpha	+
CNTN1	**Contactin-1**	**+**
CNTN2	Contactin-2	+
CNTN3	Contactin-3	
CNTN4	Contactin-4	
CNTN5	Contactin-5	+
CNTN6	Contactin-6	+
CPM	**Carboxypeptidase M**	**+**
CPO	Carboxypeptidase O	+
*DPEP1 ^EV^^,^^ EX^^,^^ S^*	***Dipeptidase 1***	***+***
DPEP2	Dipeptidase 2	
DPEP3	Dipeptidase 3	
EFNA1	**Ephrin-A1**	**+**
EFNA2	Ephrin-A2	
EFNA3	**Ephrin-A3**	**+**
EFNA4	**Ephrin-A4**	**+**
EFNA5	Ephrin-A5	+
ENPP6	Ectonucleotide pyrophosphatase/phosphodiesterase 6	+
FCGR3B	Low affinity immunoglobulin gamma Fc region receptor III-B	+
*FOLR1 ^EV^^,^^ EX^^,^^ S^*	***Folate receptor alpha***	***+***
FOLR2	Folate receptor beta	+
GAS1	Growth arrest-specific protein 1	+
GFRA1	**GDNF family receptor alpha-1**	**+**
GFRA2	GDNF family receptor alpha-2	+
GFRA3	**GDNF family receptor alpha-3**	**+**
GFRA4	GDNF family receptor alpha-4	+
GLIPR1L1	GLIPR1-like protein 1	+
GML	Glycosyl-phosphatidylinositol-anchored molecule-like protein	
GP2	**Pancreatic secretory granule membrane major glycoprotein 2**	**+**
GPC1	**Glypican-1**	**+**
GPC2	**Glypican-2**	**+**
GPC3	Glypican-3	+
GPC4	**Glypican-4**	**+**
GPC5	Glypican-5	+
GPC6	Glypican-6	+
GPIHBP1	Glycosylphosphatidylinositol-anchored high-density lipoprotein-binding protein 1	
HJV	**Hemojuvelin**	**+**
HYAL2	**Hyaluronidase-2**	**+**
IGSF21	**Immunoglobulin superfamily member 21**	**+**
ITLN1	**Intelectin-1**	**+**
IZUMO1R	Sperm-egg fusion protein Juno	
LSAMP	Limbic system-associated membrane protein	+
LY6D	**Lymphocyte antigen 6D**	**+**
LY6E	**Lymphocyte antigen 6E**	**+**
LY6G6C	Lymphocyte antigen 6 complex locus protein G6c	+
LY6G6D	**Lymphocyte antigen 6 complex locus protein G6d**	**+**
LY6H	Lymphocyte antigen 6H	
LY6K	Lymphocyte antigen 6K	+
LY6L	Lymphocyte antigen 6L	
LY6S	Lymphocyte antigen 6S	
LYNX1	**Ly-6/neurotoxin-like protein 1**	**+**
LYPD1	**Ly6/PLAUR domain-containing protein 1**	**+**
LYPD2	**Ly6/PLAUR domain-containing protein 2**	**+**
LYPD3	**Ly6/PLAUR domain-containing protein 3**	**+**
LYPD4	Ly6/PLAUR domain-containing protein 4	
LYPD5	**Ly6/PLAUR domain-containing protein 5**	**+**
LYPD6	**Ly6/PLAUR domain-containing protein 6**	**+**
LYPD6B	**Ly6/PLAUR domain-containing protein 6B**	**+**
LYPD8	Ly6/PLAUR domain-containing protein 8	
MDGA1	MAM domain-containing glycosylphosphatidylinositol anchor protein 1	+
MDGA2	MAM domain-containing glycosylphosphatidylinositol anchor protein 2	
*MELTF ^EV^^,^^ EX^^,^^ S^*	***Melanotransferrin***	***+***
MMP17	Matrix metalloproteinase-17	
MMP25	Matrix metalloproteinase-25	+
*MSLN ^EV^^,^^ EX^^,^^ S^*	***Mesothelin***	**+**
NCAM1	**Neural cell adhesion molecule 1**	**+**
NEGR1	**Neuronal growth regulator 1**	**+**
NRN1	**Neuritin**	**+**
NRN1L	Neuritin-like protein	
*NT5E ^EV^^,^^ EX^^,^^ S^*	***CD antigen CD73***	***+***
NTM	**Neurotrimin**	**+**
NTNG1	Netrin-G1	
NTNG2	**Netrin-G2**	**+**
OMG	Oligodendrocyte-myelin glycoprotein	
OPCML	Opioid-binding protein/cell adhesion molecule	+
OTOA	Otoancorin	+
*PLAUR ^EV^^,^^ EX^^,^^ S^*	***Urokinase plasminogen activator surface receptor***	***+***
PLET1	Placenta-expressed transcript 1 protein	
PRND	Prion-like protein Doppel	
PRNP	**Major prion protein**	**+**
PRSS21	**Testisin**	**+**
PRSS41	Serine protease 41	
PRSS42P	Putative serine protease 42	
PRSS55	Serine protease 55	+
PSCA	**Prostate stem cell antigen**	**+**
RAET1G	UL-16 binding protein 5	
RAET1L	UL16-binding protein 6	
RECK	**Reversion-inducing cysteine-rich protein with Kazal motifs**	**+**
RGMA	**Repulsive guidance molecule A**	**+**
*RGMB ^EV^^,^^ EX^^,^^ S^*	***Repulsive guidance molecule B***	**+**
RTN4R	**Reticulon-4 receptor**	**+**
RTN4RL1	Reticulon-4 receptor-like 1	+
RTN4RL2	**Reticulon-4 receptor-like 2**	**+**
SEMA7A	Semaphorin-7A	+
*SMPDL3B ^EV^^,^^ EX^^,^^ S^*	***Acid sphingomyelinase-like phosphodiesterase 3b***	***+***
SPACA4	Sperm acrosome membrane-associated protein 4	+
SPAM1	**Hyaluronidase PH-20**	**+**
SPRN	Shadow of prion protein	
TDGF1	Teratocarcinoma-derived growth factor 1	
TECTA	**Alpha-tectorin**	**+**
TECTB	Beta-tectorin	
TEX101	Testis-expressed protein 101	
TFPI	Tissue factor pathway inhibitor	+
THY1	**Thy-1 membrane glycoprotein**	**+**
TNFRSF10C	Tumor necrosis factor receptor superfamily member 10C	+
TREH	Trehalase	+
ULBP1	**UL16-binding protein 1**	**+**
ULBP2	**UL16-binding protein 2**	**+**
ULBP3	**UL16-binding protein 3**	**+**
UMOD	Uromodulin	+
VNN1	Pantetheinase	+
VNN2	Pantetheine hydrolase VNN2	
XPNPEP2	**Xaa-Pro aminopeptidase 2**	**+**

Table 1 displays all human proteins reported to contain a GPI linkage, which are indexed in the UniProt database. Proteins from CRC-derived EVs are highlighted in bold, while proteins identified in EVPs by proteomics in our lab are in italics (^EV^ - small EVs, ^EX^ - exomeres, ^S^ - supermeres). Vesiclepedia protein datasets were used to assess the presence of these proteins in EVs. To identify GPI-APs from CRC-derived EVs, Vesiclepedia ‘colorectal cancer cells’ datasets were scraped for the presence of GPI-Aps^[31]^.

GPI-APs are conserved from protozoa to vertebrates and play crucial roles in many physiological processes, including development, immunity, and neurogenesis^[32]^. The study of the unique structure of GPI-APs and the rich diversity of dynamic behaviors in terms of their diffusion, organization, and interactions at the cell membrane has provided important insights into how specialized domains in the cell membrane are organized, maintained, and utilized for signal transduction^[33]^. For instance, the study conducted by Brown and Rose in 1992 on GPI-anchored placental alkaline phosphatase (ALP) led to the discovery of detergent-resistant membrane (DRM) fractions that were enriched in sphingolipids, cholesterol, and GPI-APs. This work built upon the functional membrane enrichment domains proposed by Simons and van Meer in their earlier study of GPI-AP sorting and established the foundation for the lipid raft hypothesis^[33-37]^.

The process of GPI anchorage begins with the synthesis of the GPI anchor in the endoplasmic reticulum (ER). This anchor is then attached to the GPI signal sequence of the protein as a conserved post-translational modification in the ER lumen. The GPI anchor structure is then remodeled, making it act as a transport signal that actively triggers the delivery of GPI-APs from the ER to their final functional destination - the plasma membrane, extracellular media, or the endocytic/secretory membrane system - through the Golgi apparatus^[38,39]^.

### Biosynthesis and Export from the ER

**Figure 1:**
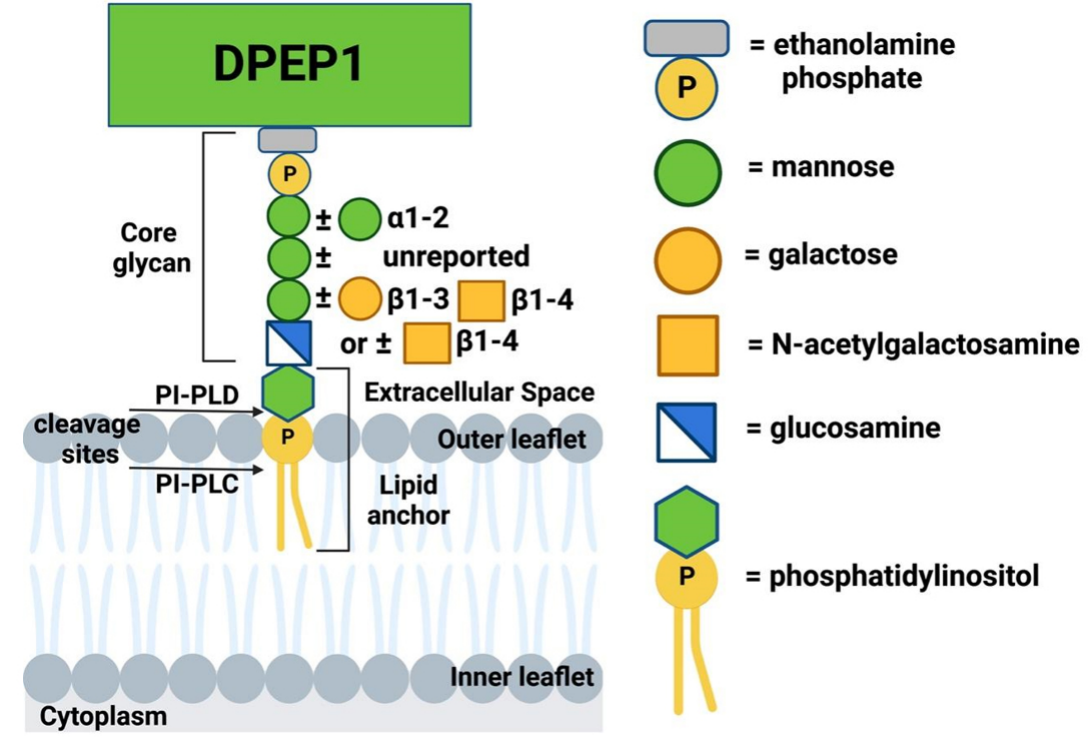
GPI-anchorage for DPEP1. DPEP1, a GPI-linked dipeptidase overexpressed in CRC, is used as an example of GPI anchorage. The exact form of phosphatidylinositol is unknown^[24]^. Glycan core additions, apart from the mannose and glucosamine backbone alone that makes up a fourth of the DPEP1 GPI anchor, are variable, with 9% of GPI anchors also having a sialic acid (not shown)^[24]^. Cleavage sites on the GPI anchor for PI-PLC and PI-PLD are shown^[40]^.

Biosynthesis of GPI-APs starts with the synthesis of GPI on the outer membrane of the ER; after synthesis of a glucosaminyl phosphatidylinositol (GlcN-PI), it is translocated to the luminal side of the ER by a yet unknown “flippase”^[26]^. On the luminal side, the GPI is sequentially processed by multiple proteins to yield the mature precursor that serves as an anchor for protein attachment [Figure 1].

**Figure 2:**
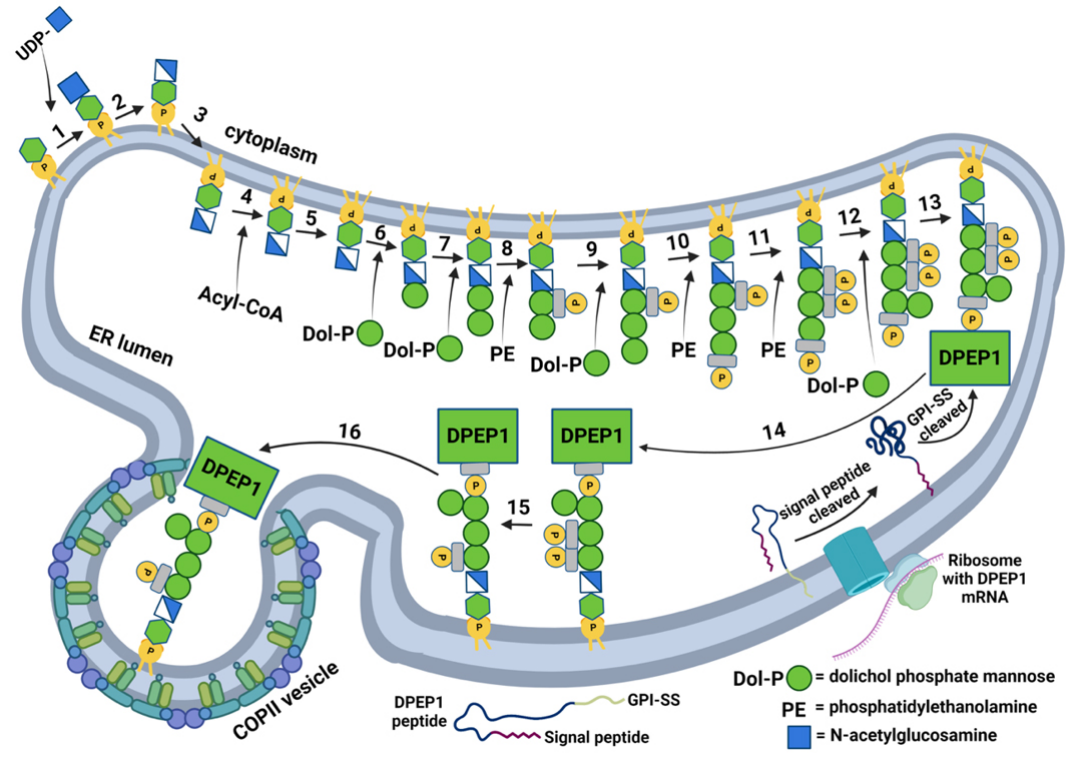
GPI anchor biosynthesis in the ER. Steps for GPI anchor biosynthesis and protein anchoring are shown^[44]^. UDP denotes uracil diphosphate, PE denotes phosphatidylethanolamine, signal peptide denotes an N-terminal signal peptide that is not required for GPI anchorage, and GPI-SS denotes the GPI signal sequence that may be associated with the membrane before cleavage^[45,46]^. Step 3 involves an unknown mechanism that allows for the movement of the phosphatidylinositol to the luminal side of the ER. Step 4 involves the addition of a fatty acid chain, while Step 5 involves lipid remodeling. Step 13 involves the cleavage of the GPI-SS and the addition of the protein onto the preassembled GPI anchor. The anchor is further modified and is released from a COPII-coated vesicle for trafficking to the Golgi.

This extensively studied process requires about 20 distinct gene products involved in the sequential addition of monosaccharides to phosphatidylinositol^[16,26]^. The mature GPI anchor precursor is then attached in a single step to newly synthesized proteins that contain a GPI signal sequence (GPI-SS) at their C termini; this attachment is executed by a GPI-transamidase complex located in the ER lumen [Figure 2]. Immediately after attachment to the protein, remodeling enzymes modify both lipid and glycan portions of the GPI anchor to convert it into a transport signal that actively promotes ER export^[41]^. Interestingly, some GPI-transamidase complex subunits are upregulated in cancer; for example, the PIG-U subunit is upregulated in CRC, as well as PIG-T and PIG-K^[18,42]^. Moreover, the blockade of GPI remodeling in the ER is being studied as a therapeutic intervention in cancer^[43]^. Upregulation of GPI-transamidase complex subunits might allow for the increased presence of GPI-linked proteins at the membrane of CRC cells and thus more turnover and potential incorporation into sEVs. Further investigation of GPI trafficking machinery may provide novel insights into the roles of GPI-APs in CRC EVs and nanoparticles.

Correctly folded and assembled secretory proteins are packaged into protein-coated vesicles for transport from the ER to the Golgi. The vesicles are formed through the polymerization of the cytosolic coat protein complex II (COPII) at specific ER membrane domains called ER exit sites (ERESs)^[26]^. COPII coating actively captures and concentrates most secretory proteins at ERESs for packaging into COPII vesicles^[47]^. In mammalian cells, GPI-APs are incorporated into the same ERESs and COPII vesicles as other proteins during their exit from the ER for delivery to the Golgi^[48]^. The concentration of GPI-APs at ERESs is dependent on the p24 complex^[49]^. Although this process has only been described in yeast, it is likely that the mammalian p24 complex recognizes GPI-APs in a similar way^[38]^.

### Arrival to Golgi and post-ER Quality Control

**Figure 3:**
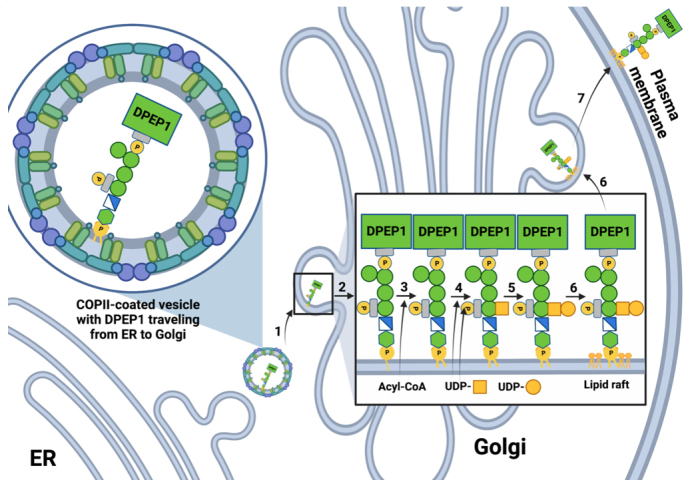
GPI anchor biosynthesis in the Golgi and trafficking to the plasma membrane. Steps for GPI anchor modification and trafficking from the ER to the plasma membrane are shown^[44]^. The yellow box denotes an N-acetylgalactosamine, whereas the yellow circle denotes a galactose. GPI-APs preferentially reside in lipid raft-rich areas, as is depicted both in the Golgi and at the plasma membrane.

Upon reaching the Golgi, the remodeled GPI-APs are thought to dissociate from the p24 complex^[39]^, a claim supported by the finding that only the ER form of GPI-APs is found associated with p24 proteins^[50]^. This dissociation is believed to be caused by a decrease in pH between the ER and Golgi, which induces conformational changes in the p24 complex and, therefore, the binding affinity of the p24 complex for GPI-Aps^[49]^. Once released, the remodeled GPI-APs can continue through the secretory pathway and be finally delivered to the plasma membrane [Figure 3].

The ability of a GPI anchor to concentrate proteins into membrane domains aids in its sorting along the entire secretory pathway^[51]^. The sorting in mammalian cells primarily occurs at the Golgi^[52,53]^, with the sorting process preceded by the post-translational modification of the lipid tail so that GPI-anchored proteins are incorporated into specific membrane domains and transported efficiently to the cell surface^[38]^. After being fully glycosylated during their passage through the Golgi cisternae, GPI-APs exit the Golgi from the trans-Golgi network (TGN) in secretory vesicles that transport them to the plasma membrane^[38]^.

**Figure 4:**
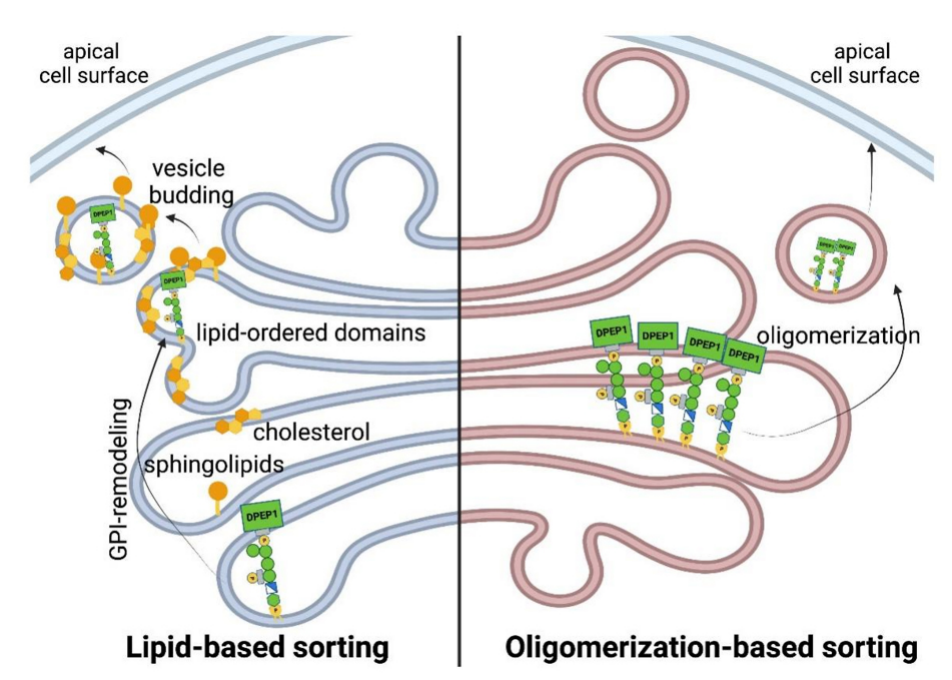
GPI-APs can be sorted to apical cell surface through two distinct mechanisms. The left side depicts the lipid-based sorting mechanism in the Golgi, where remodeling of GPI-APs allows for clustering into lipid-ordered domains that leads to apical sorting. The right side depicts the oligomerization-based sorting mechanism in the Golgi, where oligomerization of GPI-APs through ectodomain interactions allows for clustering and sorting to the apical cell surface.

Sorting of GPI-APs to the cell surface is a crucial step in the proper localization and function of these proteins. In polarized cells like neurons or epithelial cells, GPI-APs are predominantly sorted to the axon or to the apical domain, respectively^[54-57]^. In most polarized epithelial cells, the GPI anchor appears to act as an apical sorting signal, as most GPI-APs are delivered in specific secretory vesicles from the TGN to the apical but not to the basolateral cell surface^[58]^. Several mechanisms facilitating apical sorting have been described in the literature, with the main ones being lipid-based sorting and oligomerization-based sorting [Figure 4]. The lipid-based sorting mechanism suggests that remodeling of the GPI-lipid in the Golgi causes GPI-APs to cluster and associate with sphingolipids and cholesterol, facilitated by the two saturated fatty acids^[59]^. These specialized lipid-ordered domains then serve as selective platforms for vesicle budding at the TGN^[35,51]^. Lipid-based sorting is based on the observation that the sorting correlates with the acquisition of two saturated fatty acids by the GPI anchor through GPI-lipid remodeling in the Golgi, which leads to the recovery of GPI-APs with DRMs^[52,53]^ and the fact that the apical membrane is enriched in saturated lipids such as sphingolipids, which are made in the Golgi, and cholesterol^[37,60]^. Supporting this lipid-based sorting mechanism, inhibitors of sphingolipid biosynthesis and/or removal of cholesterol have been shown to impair the apical sorting of GPI-Aps^[61,62]^. In contrast, another report has shown that the lipids of GPI-APs that have not been remodeled in the Golgi can still be transported to the plasma membrane with the same efficiency as remodeled GPI-APs with saturated fatty acids^[63]^.

These reports spurred the search for alternative pathways that may also regulate apical targeting such as oligomerization of GPI-APs, which has also been suggested as a key factor in the apical sorting of GPI-APs. GPI-APs are known to form high molecular weight complexes during their transport to the apical membrane, and this process of oligomerization is crucial for proper sorting to the apical domain. Impairment of this oligomerization leads to the missorting of GPI-APs to the basolateral domain^[62,64]^. Interestingly, some specific GPI-APs under control conditions do not oligomerize and are basolaterally sorted, but the addition of cholesterol to cells is sufficient to drive the oligomerization and consequent apical sorting^[65]^. Indeed, it has been shown that oligomerization depends on cholesterol in polarized epithelial cells and requires fatty acid remodeling in nonpolarized cells, like fibroblasts^[62]^. Furthermore, the process of oligomerization is dependent on protein-protein interactions through the ectodomains of GPI-APs and has been shown to facilitate the segregation of GPI-APs from other protein classes^[65]^. It is also believed to promote the coalescence of small lipid domains into larger, more stable domains, thereby favoring vesicle budding from the TGN^[62]^. Additionally, in polarized epithelial cells and during the loss of polarity, the mechanism of oligomerization-based sorting of GPI-APs in the Golgi becomes of great physiological importance, as it controls both their organization and function at the apical membrane^[66]^.

Several other factors play a role in the apical sorting of GPI-APs. N-glycosylation has been shown to be required for apical delivery of GPI-APs, which suggests a potential involvement of galectins^[67]^. Accessory factors such as MAL/VIP17, annexins, flotillins, and stomatin have been proposed to contribute to apical sorting by promoting clustering of GPI-APs and other apically targeted proteins in lipid domains, but the underlying mechanisms have not been elucidated^[68-73]^.

Nevertheless, the GPI anchor does not always serve as an apical sorting signal, as some GPI-APs in different epithelial cell lines are sorted and transported basolaterally^[74,75]^. In MDCK cells, GPI-APs are primarily found on the apical surface, whereas in Fischer rat thyroid cells, they are sorted to the basolateral domain^[74]^. Furthermore, the sorting of a protein as apical or basolateral can vary even within the same cell line. For example, in MDCK cells, some GPI-APs such as PLAP are primarily sorted to the apical surface, while others such as PrP are trafficked to the basolateral domain^[62,75,76]^. The basolateral sorting of these GPI-APs appears to be more dependent on protein oligomerization rather than lipid-based sorting. DRM association was observed for both apical and basolateral GPI-APs; however, only apically localized GPI-APs formed high molecular weight complexes^[62]^. An especially interesting case is the GPI-anchored high-density lipoprotein-binding protein 1 (GPIHBP1), which transports lipoprotein lipase from subendothelial spaces to the luminal face of capillary endothelial cells and is enriched in both the basolateral and apical plasma membrane domains of these cells^[77]^. The mechanism behind basolateral sorting of GPI-APs is not well understood and remains a subject of ongoing research. This lack of understanding becomes even more pronounced when examining cases of loss of cell polarity, where the normal sorting patterns of GPI-APs are disrupted.

### Release from the Cell Membrane

For many GPI-anchored proteins, reaching the plasma membrane is not the final destination. Shortly after the first biochemical identification and structural characterization of GPI anchors in eukaryotic cells, it was proposed that one of the major physiological roles of GPI anchorage of cell surface proteins may relate to constitutive and/or controlled release of the protein moiety into the extracellular space^[78-80]^. Cell- surface GPI-APs are released by GPIase activity in many crucial biological events, such as cellular proliferation, development, neurogenesis, and reproduction^[32]^. This process has significant implications not only from a biological perspective, but also from a clinical standpoint. For example, circulating GPI-APs, such as CEAMCAM5 and TDGF1, can serve as clinical biomarkers of disease^[81-83]^.

Several pathways mediate the release of GPI-APs from the plasma membrane by vesiculation or cleavage. Currently, the predominant mechanism for the majority of GPI-APs is believed to be lipolytic cleavage, primarily by (Glyco)phosphoinositol-specific phospholipases, also known as (G)PI-PLs. A variety of phospholipases with cleavage specificity C or D(GPI-PLC/D) have been detected, which manage to separate the protein moiety and GPI anchor of GPI-AP in cell-free or cellular test systems. (G)PI-specific PLC [(G)PI-PLC] and GPI-specific PLD (GPI-PLD) cleave the GPI anchor at different sides of the phosphodiester bond within PI^[40]^. The bond between the phosphate and glycerol residues is cleaved by (G)PI-PLC, whereas the bond between the inositol and phosphate residues is cleaved by GPI-PLD^[78,84,85]^. However, it has been challenging to identify the responsible enzymes that act as GPIases. Some well-characterized GPIases to date include GPI-PLD, NOTUM, GDE2, and ACE, but these do not encompass all the GPI-PLCs needed for mammalian GPI-AP cleavage from the cell membrane^[32,78]^. GPI-PLD, encoded by the gene GPLD1, was initially identified as a human serum protein^[58,86]^, and its biochemical^[87,88]^ and molecular^[89]^ features have been extensively characterized since its discovery. It is a soluble protein with two functional domains, an N-terminal catalytic domain and a predicted C-terminal β propeller domain^[90,91]^. Membrane-bound GPI-APs, such as PLAUR^[92,93]^, CEACAM5^[94]^, PRSS8^[95,96]^, and TDGF1^[97,98]^, are released from the cell surface by GPI-PLD and have roles in several important cellular processes, such as adhesion, differentiation, proliferation, survival, and oncogenesis^[32,95]^.

While GPI-APs can be released from the cell surface via phospholipases, they also can be found extracellularly attached to lipids with an intact GPI anchor. The modes of release for an intact GPI anchor include release via 1) vesicles with intact GPI-APs attached to the vesicular membrane, 2) particles with GPI anchors attached to particles’ phospholipid monolayer, and 3) multimers or micelle-like complexes with GPI-APs bound to the hydrophobic cleft of carrier proteins or assembled with phospholipids and cholesterol of the micelle-like complexes^[78]^. These methods of release are relevant to our studies as they result in GPI-APs as cargo from secreted vesicles and nanoparticles. Along with the biogenesis, sorting, and release of GPI-APs informing the EV field, the identification of actual GPI-AP cargoes that are released from cells is critically important as they confer functions to EV and nanoparticle subsets.

### Secreted GPI-APs Relevant to CRC

**Figure 5:**
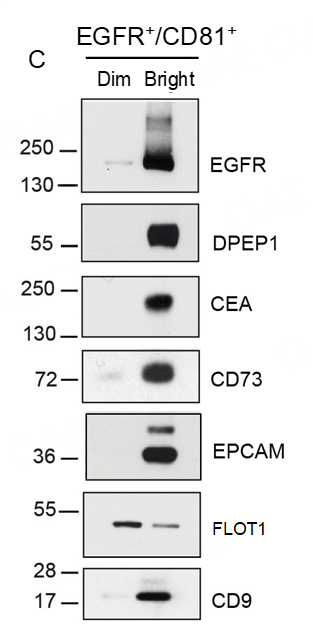
GPI-APs are preferentially enriched in classical exosomes with EGF receptor (EGFR) from DiFi cells. Fluorescence-activated vesicle sorting (FAVS) was performed on sEV pellet using directly-labeled antibodies to EGFR and the tetraspanin CD81. EGFR and CD81 double-positive vesicles were sorted into bright and dim populations. Immunoblotting shows marked enrichment of EGFR, DPEP1, the known CRC biomarkers CEA (CEACAM5) and EPCAM, and CD73, a GPI-linked ectonucleotidase that converts 5’AMP to adenosine, a known T cell immunosuppressant.

Our interest in GPI-APs on EVs and nanoparticles was sparked by discoveries we reported in *Nature Cell Biology*^[13]^. We were especially intrigued by the results of our fluorescence-activated vesicle sorting (FAVS) analysis of exosomes isolated from a CRC cell line, DiFi. DiFi cells have been chosen for benchmarking studies in Phase 2 of the Extracellular RNA Communication Consortium (ERCC2), which will consequently lead to a greater understanding of CRC EV communication and could lead to greater insights involving GPI-Aps^[99]^. Individual sEVs were flow sorted with directly labeled antibodies to the classical exosome tetraspanin, CD81, and EGFR. We found there was marked enrichment for the GPI-APs DPEP1, CD73, and CEACAM5 in the CD81/EGFR double-bright exosome population compared to the CD81/EGFR double-dim population [Figure 5]^[13]^.

**Figure 6:**
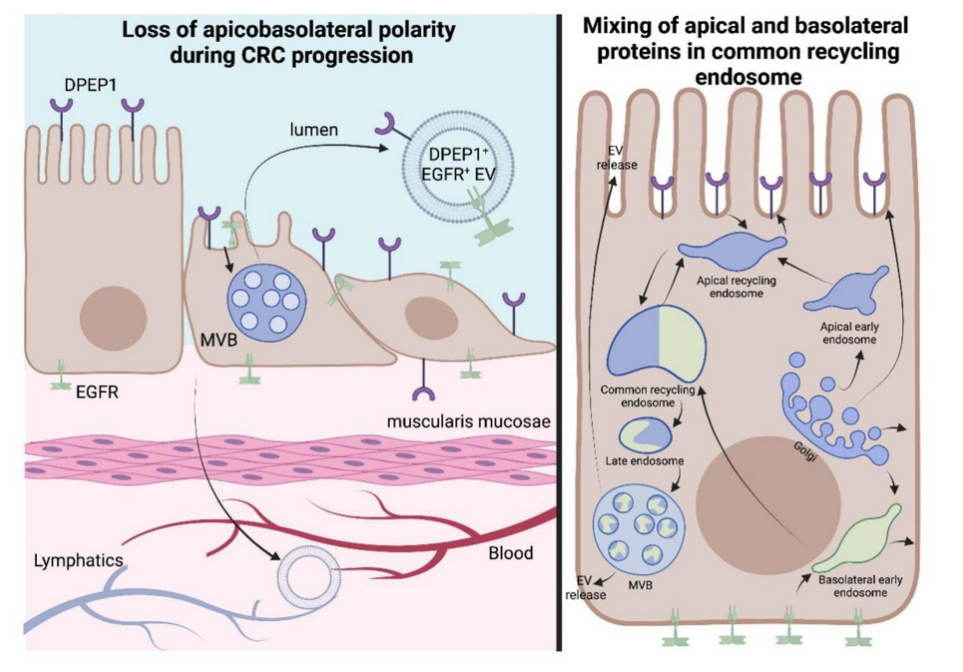
Two methods of sorting apical and basolateral proteins into the same EV. The left side depicts the progressive loss of polarity during CRC progression, leading to apical (DPEP1) and basolateral (EGFR) cell-surface proteins being in the same EV. The right side depicts different routes of trafficking of apical and basolateral proteins, which can lead to their presence in the common recycling endosome^[104]^. This endosome can then mature into an MVB, which would contain both DPEP1 and EGFR in the same intraluminal vesicle.

In fact, DPEP1 was more abundant than EGFR by mass spectrometry^[13]^. Further profiling of sEVs, exomeres, supermeres, and nonvesicular fractions revealed an array of GPI-APs enriched in extracellular fractions in comparison to DiFi cells, which reflects a subset of the GPI-APs reported to be found in CRC EVs [Table 2]^[13]^. Some GPI-APs are uniquely detected in the exosome fraction compared to exomeres/supermeres, while others are simply a highly abundant protein that is enriched in one fraction in comparison to others. The heterogeneity and biogenesis of EV subpopulations remain to be a focus of the field, and our results pose yet another unsolved question: how do apical (DPEP1) and basolateral (EGFR) proteins become presented on the same EV? One possibility is a loss of apico-basolateral polarity that is characteristic of poorly differentiated CRCs [Figure 6]. Another possibility is that endosomal pathways from the apical and basolateral sides converge, allowing for mixing of apical and basolateral proteins in a common recycling endosome so that both protein subsets are present in the same MVB and released in the same exosome[Figure 6]. While it is reported that recycling endosomes actively sort protein cargos into subdomains to ensure apical and basolateral polarity, that may not apply to MVBs as one study has shown that EVs released from a CRC cell line-derived organoid can have both apical and basolateral proteins^[100,101]^. Figure 6 (right) also depicts the trafficking routes that synthesized GPI-APs can take in order to be incorporated into sEVs. There are myriad of signaling pathways that can allow for cargo sorting into EVs as well as vesicle formation, which have been previously described in other reviews^[102,103]^. With these considerations in mind, we will now highlight a subset of these GPI-APs that were identified from our proteomic data and discuss their possible functional roles in CRC.

**Table 2:** Comparison of exosomes, exomeres, and supermeres

**Extracellular particle type:**	**Exosomes**	**Exomeres**	**Supermeres**
Size	< 200 nm	~ 35 nm	~ 25 nm
Biogenesis	Endosomal origin	Unknown	Unknown
Abundant proteins	CD9, CD63, CD81, Alix, Syntenin-1	ENO1, GANAB	TGFβi, AGO2, ACE2, PCSK9
GPI-APs	DPEP1, CD73, MELTF, CEACAM5, SMPDL3B, PLAUR, CEACAM6, CD55, CD59 FOLR1	DPEP1, CD73, CEACAM5, CEACAM6, CD59	DPEP1, CD73, MELTF, CEACAM5, CEACAM6, CD59
RNA content abundance	++	+	+++
RNA species abundance in comparison to cells	rRNA, lncRNA	miRNA, snRNA	Enriched for snRNA, miRNA, yRNA
Lipid content	Lipid bilayer	little	little
Functional properties	Various functions, many reported in previous reviews	Transfer lactate and cetuximab resistance, liver effects, tumor growth by AREG transfer, transfers ST6Gal-I	Transfer lactate and cetuximab resistance, Crossing the blood-brain barrier, liver effects

Table 2 shows distinguishing characteristics among EVs and nanoparticle subsets^[2,3,12-14,170]^. GPI-AP data curated from recent supermere publication^[13]^. Of the 140 GPI-APs recognized by Uniprot, 10 were identified by proteomics in extracellular fractions isolated from DiFi cells^[13]^.

### DPEP1

As noted above, the most abundant protein found in sEVs from the CRC line, DiFi, was DPEP1^[13]^. DPEP1 was originally reported as an enzyme in kidney epithelial cells with dipeptidase activity^[105]^. Subsequently, Vogelstein et al. identified *DPEP1* as a potential biomarker for CRC as it met their criteria of being a cell surface or secreted protein that was upregulated more than 20-fold in adenomas and CRCs^[106]^. This upregulation of DPEP1 in CRC has been confirmed by other groups, further spurring interest in studying DPEP1 in a CRC context^[107,108]^. DPEP1 has been shown to have roles in proliferation, invasion, metastasis, and drug resistance^[109-112]^. The few scant reports on DPEP1 in EVs have noted its presence in kidney or urine EVs, although one study reported that it was enriched in metastatic CRC EVs^[113-115]^. We focused on characterizing DPEP1 biochemically and in relation to EV subpopulations since it was the most abundant protein in sEVs^[13]^. We found that DPEP1 staining was largely absent in normal colon^[13]^. Clinically well-annotated tissue microarrays of CRCs were scored for DPEP1 immunoreactivity by an experienced gastrointestinal pathologist^[13]^. DPEP1 staining was observed in the majority of CRCs and diffuse cytoplasmic staining for DPEP1 was associated with a worse progression-free and overall survival^[13]^. DPEP1 was found to be enriched specifically in EVs in comparison to nanoparticles and nonvesicular fractions, especially in CD81/EGFR double-bright, flow-sorted exosomes, along with other GPI-APs of interest, including CD73 and CEACAM5 [Figure 5]^[13]^. The EV-bound DPEP1 was found to be α2, 6-sialylated and colocalized with CD63 in MVBs, providing strong evidence for its endosomal biogenesis^[13]^. DPEP1-positive EVs and DPEP1/CEACAM5 double-positive, flow-sorted EVs were increased in the plasma of CRC patients in comparison to healthy controls. Taken together, these findings warrant monitoring EV DPEP1 levels in the plasma of CRC patients as a non-invasive biomarker.

No studies on the function of EV-bound DPEP1 have been reported, but there is a growing body of literature concerning a new nonenzymatic function of DPEP1, that is, binding neutrophils and monocytes^[116,117]^. These reports show that DPEP1 can be expressed by endothelial cells in response to an inflammatory insult, resulting in increased neutrophil and monocyte binding and infiltration^[116,117]^. Interestingly, both the enzymatic inhibitor and neutrophil-binding inhibitor of DPEP1 reduce immune cell recruitment and binding, suggesting that DPEP1 might have a role in producing a chemoattractant through its enzymatic activity^[117]^. Our future studies will involve determining if DPEP1 expressed on CRC cells can interact with neutrophil and monocytic populations and how DPEP1-positive EVs might enhance this interaction or act as decoys, as we have shown occurs with ACE2-containing EVs in a COVID-19 setting^[118]^.

### CD73

Along with DPEP1, one of the GPI-APs enriched in CD81/EGFR double-bright exosomes was CD73. CD73 is a 5’-ectonucleotidase that converts extracellular AMP into immunosuppressive adenosine with similarities to DPEP1 in that it also acts as an adhesion factor between lymphocytes and endothelial cells^[119,120]^. This GPI-AP has been shown to be upregulated across many cancer types, and it has roles in immune evasion, angiogenesis, proliferation, migration, and invasion^[121]^. In contrast to DPEP1, mechanistic studies regarding CD73-positive EVs in cancer are more established, including a report that CD73 is enzymatically active in B cells, although few studies are directly related to CRC^[122]^. Cancer-derived CD73-positive vesicles have been implicated in T-cell suppression, angiogenesis, resistance to anti-PD-1 therapy, and suppression of T-cell clonal expansion^[19,123-125]^. In a recent report, EVs isolated from a CRC cell line, DLD-1, as well as two other cancer lines, H292 and OvCAR3, were found to be enriched for CD73 compared to the cells themselves^[126]^. A bispecific antibody to CD73 and EpCAM not only reduced the CD73 activity of these cancer-derived EVs, but also inhibited the effects of CD73 on reducing T-cell proliferation and rescued the anti-tumor properties of T-cells^[126]^. While a number of clinical trials have been conducted or are under way for inhibition of CD73, the full potential of therapeutics might not be reached without understanding how CD73^+^ EVs contribute to cancer progression^[119,127]^.

We began to consider CD73-containing EVs as a biomarker for CRC when we discovered that they were enriched with other GPI-APs in classical exosomes isolated from CRC cells^[13]^. We found that CD73, like DPEP1, was α2, 6-sialylated and that CD73 immunoreactivity was increased in CRC tissue compared to normal colonic epithelium^[13]^. CRC patients generally had more CD73 in sEVs isolated from plasma than normal individuals, and, more broadly, CD73 was enriched in sEVs from a variety of different cancer cell lines, strengthening its utility as a pan-cancer biomarker^[13]^. Future studies along this line of investigation might involve measuring the ability of CD73-positive EVs released from CRC tumors to produce adenosine and testing the efficacy of CD73 inhibitors against circulating EVs, as systemic immunosuppression might affect metastatic ability that could not be achieved by only tumoral CD73.

### CEACAM5/CEACAM6

CEACAM5, typically referred to as CEA, is a commonly used CRC biomarker. Both CEACAM5 and CEACAM6 are GPI-APs in the carcinoembryonic antigen (CEA) family, so called because they were originally believed to be expressed in fetal development, absent in healthy adults, but expression reappearing as cancers revert to an onco-fetal state^[128,129]^. Functionally, CEACAM5 reportedly acts as an adhesion molecule, as well as having roles in inhibiting apoptosis, cell polarization, and differentiation in CRC cells, as well as increasing metastatic ability^[130-133]^. CEACAM6, sometimes referred to as CD66c, has similar roles, with reports highlighting its role in CRC growth and immunosuppression of T cells; its expression as assessed by IHC in tissue is touted as a poor prognosis marker in CRC^[129,131-135]^.

Monitoring plasma CEA levels following surgical resection of the tumor continues to be used as a biomarker for CRC recurrence, although its reliability is questioned^[136]^. The field has pivoted to multimarker analysis to increase the reliability and sensitivity of CEA’s diagnostic and prognostic value, including immune population ratios, cytokines, or other cancer antigens, with some improvement in predicting recurrence or disease presence^[137-140]^. While measuring CEA levels alone in the plasma or in combination with other markers has been a focus for increasing biomarker accuracy and sensitivity, the EV community has begun to see merit in isolating EV-bound CEA proteins for diagnostic and prognostic value. CEACAM6 was detected from a CRC line, LIM1215, in EVs, and has been reported to be a general marker for neutrophil-derived EVs^[141]^. One study has shown that exosomal CEA from plasma increased sensitivity and specificity for predicting distant metastasis, while another has shown that CEA^+^ microvesicle levels could distinguish between benign polyps and CRC^[142,143]^. Along with using EV isolation to increase the predictive value of CEA as a biomarker, groups have been combining non-EV bound CEA levels in combination with other biomarkers that are present on EVs or found other markers that have higher sensitivity. TSPAN1-positive exosomes by CD63 capture were found to have higher sensitivity in detection of CRC than plasma CEA levels alone^[144]^. CPNE3 combined with CEA on EVs was a superior diagnostic biomarker for CRC than either protein alone^[145]^. Other exosomal components or molecules associated with EVs that increased diagnostic accuracy when combined with CEA include miR-150-5p, lnRCA CRNDE-h, and CAT1^[146,147]^. We utilized FAVS to flow sort EVs double-positive for CEA and DPEP1 from the plasma of CRC patients as a means of detecting EVs released from the cancer rather than other sources^[13]^. CEACAM5 was present in exomeres and supermeres isolated from CRC cell lines and detected in the plasma from CRC patients but not from healthy controls^[13]^. Interestingly, EVs from bacteria can affect the release of CEA. Exposure of EVs isolated from *Lactobacillus rhamnosus* but not from other bacteria to CRC cell lines increased CEA levels and inhibited cell proliferation^[148]^. Understanding how the microbiome and associated EVs might affect both CRC progression and detection is an emerging field of study.

### GPC1

Glypican-1, encoded by the gene *GPC1*, is another GPI-AP that we identified as being secreted by a wide variety of cancer cell lines, as well as primary kidney epithelial cells, with a marked enrichment in exomeres and supermeres in comparison to sEVs^[13]^. Glypican-1 can modulate signaling pathways by binding to growth factors, and it has been reported to regulate TGF-β signaling to increase proliferation and migration while inhibiting apoptosis in CRC cells^[149,150]^. Glypican-1 has been reported to be a cancer exosomal marker, in contrast to our finding that they are mostly associated with nanoparticles^[151]^. In a CRC context, overexpression of Snail, a transcription factor involved in EMT, has been reported to increase the presentation of glypican-1 on CRC EVs^[152]^. Glypican-1 has been shown to be increased in EVs isolated from CRC tumor tissue and plasma in comparison to normal controls and was regulated by miRNAs, leading to reduced secretion in an EV-bound form^[153]^. The discrepancy between our results showing that glypican-1 is enriched in nanoparticles, not sEVs, highlights the ever-evolving EV field and the importance of continually improving methods of isolation and parsing subsets^[13]^.

### Challenges and Future Directions

Previous findings from our laboratory, and others, have reported the enrichment of GPI-APs in CRC sEVs^[5,13,154]^. The presence of GPI-APs in sEVs dates back to the early 1990s, at which time several GPI-APs, including AChE, DAF, MIRL, and LFA-3, were identified in human reticulocyte sEVs^[155]^. Since then, GPI-APs have become recognized as part of the standard repertoire of sEVs, and functional roles assigned to them^[78,156,157]^. For instance, NKG2D-ligands including GPI-APs such as ULBP1, ULBP2, and ULBP3 have been reported to display two main mechanisms of release: shedding by metalloproteases and recruitment to sEVs. Expression of these proteins is induced by stress and signals for immune activation, and they can also serve as immune decoys when released by tumor cells^[158]^. The enrichment of lipid rafts and sphingomyelins in sEVs make them a perfect carrier for GPI-Aps, as lipid-based sorting is a main mechanism for GPI-AP delivery to the cell surface^[7,159,160]^. GPI-APs can be incorporated into early endosomes that will eventually mature into multivesicular bodies (MVBs) or as newly synthesized GPI-APs that can be directly trafficked from the Golgi to MVBs^[161]^. While there are multiple ways that GPI-APs can be packaged into exosomes^[78,161]^, it is believed that the majority of GPI-APs leaving cells via exosomes start their journey at the cell surface. Furthermore, this protein class has been associated with exosome biogenesis; for example, the abundance and activity of the GPI-AP, tetherin, affects the ability of exosomes to detach from the donor cell membrane and adhere to the recipient cell membrane. In tetherin knockout cells, there was a reported decrease in vesicles that remain attached to the plasma membrane clustered at its surface after MVB fusion with the plasma membrane and an increase in exosomes discharged in the medium. This phenotype could be rescued by wild-type tetherin but not tetherin lacking its GPI anchor^[162]^.

Going forward, the forms and compartments in which extracellular GPI-APs are found will be informative. Although there is limited published information on GPI-APs in exomeres and supermeres due to their recent discovery, GPI-APs are transported in various non-vesicular structures in the extracellular space, both as particles and multimers. The absence of an EV membrane dictates the need for other ways of shielding the GPI anchor from the aqueous milieu, such as embedment in phospho (mono- or bi-)layers surrounding non-vesicular lipid-filled particles (like surfactant-like particles, milk fat globules, nodal vesicular particles, lipoprotein-like particles), oligomerization or multimerization without the use of additional constituents, or assembly into heterometric structures with specific carrier or scaffolding proteins^[78,163-165]^. Lipid-based sorting appears to indicate various methods for sorting GPI-APs into EVs, for both releases with an intact GPI anchor, as well as cleavage of the GPI anchor. Future lipidomic analysis of exomeres and supermeres might offer insights into their ability to include intact GPI-APs based on the lipid content. There are, however, other ways of secreting GPI-APs aside from having an intact GPI anchor inasmuch the anchor can be cleaved off both intracellularly and extracellularly. Intracellular cleavage might favor incorporation into exomeres and supermeres as they are released particles with other proteins reminiscent of stress granules and this pathway might inform the ways these nanoparticles form and mature. In contrast, extracellular cleavage could inform EVP interplay in the circulation, which is especially important in the context of the tumor microenvironment. Indeed, some of the GPI-APs that have been identified in sEVs and supermeres, such as PLAUR and CEACAM5, have been shown to be cleaved by GPLD1^[32,92-94]^. Furthermore, the effects of extracellular cleavage of GPI-APs by GPI-PLD, which is abundant in serum, and the accessibility for cleavage provided by the inclusion of GPI-APs in different types of EVPs, are important when considering specific GPI-APs as biomarkers^[166]^.

Another area where the study of EVPs and GPI-APs can complement each other is the apical versus basolateral sorting of GPI-APs. The impact of oligomerization, missorting, and differential sorting of different forms of the same protein (isoforms, glycosylated forms) can inform the biogenesis of EVPs and, in turn, increase our understanding of the underlying mechanisms of apical/basolateral sorting of GPI-APs. The heterogeneity of exosomes, and specifically heterogeneity between apically and basolaterally secreted exosomes from polarized cells, has been discussed in the literature. Some authors claim that no less than 30% of the total protein cargo is different between the two fractions^[167]^. Moreover, protein cargo in the apical EV fraction has been reported to be more homogeneous compared to its basolateral counterpart^[3]^. Moreover, as stated above, we have been able to isolate and sort individual exosomes with characteristic apical and basolateral cargo, which provides a unique opportunity to understand mechanisms of biogenesis in the context of loss of polarity [Figure 6]. Some of the proteins implicated in the apical sorting of GPI-APs, such as flotillins and annexins, are accepted as characteristic sEV marker proteins^[168]^, but have been shown to facilitate ESCRT-independent exosome pathway (flotillins^[169]^), and are marker proteins of EVs distinct from classical exosomes (annexins A1 and A2^[2]^).

Along with providing insights into the biogenesis of EVPs and the secretion patterns of a cell, the study of GPI-APs allows for assigning functions to extracellular carriers based on cargo. Our lab has previously shown that the cargo, ACE2, on sEVs and exomeres can act as a decoy for SARS-CoV-2 virus^[118]^. Assigning function to an extracellular particle based on its cargo can be translated to the CRC field in relation to GPI-APs, as we have identified a number of GPI-APs present in sEVs, exomeres, and supermeres [Table 2]. One striking example is that CD73 is enriched in classical exosomes^[13]^. As discussed above, CD73-positive EVs that aid in adenosine production can alter the TME toward an immunosuppressive state^[126]^. It will be interesting to determine the functions of the newly discovered supermeres and exomeres based on their cargo^[13]^.

In conclusion, with the rapid evolution of the EVP field and the emergence of new extracellular particles that offer the ability to allocate cargo previously thought to be in EVs to their proper carrier, approaches taking into account specific protein families like GPI-APs abundant in EVs might offer some help in overcoming EVs notorious heterogeneity. Moreover, studying these proteins in the context of exosomes, exomeres, and supermeres might inform us about EVP biogenesis, trafficking and release. In this context, like in the past GPI-APs might offer especially valuable fundamental insight, as was the case with understanding the membrane organization and the discovery of lipid rafts. However, given the broad adoption of GPI-APs into EVPs as cargo, and certain GPI-APs promising potential as biomarkers and therapeutic targets for CRC, this direction of investigations has the capacity to translate into significant clinical applications, including the development of new diagnostic tools and therapeutic strategies.

## DECLARATIONS

### Copyright

© The Author(s) 2023.
